# Extracellular enzymes secreted in the mycelial block of *Lentinula edodes* during hyphal growth

**DOI:** 10.1186/s13568-023-01547-6

**Published:** 2023-04-25

**Authors:** Nanae Kobayashi, Nagisa Wada, Haruna Yokoyama, Yuki Tanaka, Tomohiro Suzuki, Naoto Habu, Naotake Konno

**Affiliations:** 1 School of Agriculture, Utsunomiya University, 350 Mine-machi, Utsunomiya, 321-8505 Tochigi Japan; 2 Graduate School of Regional Development and Creativity, Utsunomiya University, 350 Mine-machi, Utsunomiya, 321-8505 Tochigi Japan; 3 Center for Bioscience Research and Education, Utsunomiya University, 350 Mine-machi, Utsunomiya, 321-8505 Tochigi Japan

**Keywords:** *Lentinula edodes*, Laccase, Mushroom, Sawdust medium cultivation, White-rot fungi

## Abstract

*Lentinula edodes* (shiitake mushroom) is one of the most widely cultivated edible mushrooms and is primarily cultivated using sawdust medium. While there have been improvements in the cultivation technology, the mechanism of mycelial block cultivation, such as mycelial growth and enzymatic sawdust degradation, has not been clarified. In this study, the mycelium was elongated longitudinally in the bottle sawdust culture for 27 days, and the cultivated sawdust medium was divided into three sections (top, middle, and bottom parts). To determine spatial heterogeneity in the enzyme secretion, the enzymatic activities of each part were analyzed. Lignocellulose degradation enzymes, such as endoglucanase, xylanase, and manganese peroxidase were highly secreted in the top part of the medium. On the other hand, amylase, pectinase, fungal cell wall degradation enzyme (β-1,3-glucanase, β-1,6-glucanase, and chitinase), and laccase activities were higher in the bottom part. The results indicate that the principal sawdust degradation occurs after mycelial colonization. Proteins with the laccase activity were purified from the bottom part of the medium, and three laccases, Lcc5, Lcc6 and Lcc13, were identified. In particular, the expression of Lcc13 gene was higher in the bottom part compared with the level in the top part, suggesting Lcc13 is mainly produced from the tip region and have important roles for mycelial spread and nutrient uptake during early stage of cultivation.

**Supplementary Information:**

The online version contains supplementary material available at 10.1186/s13568-023-01547-6.

## Introduction

*Lentinula edodes* (shiitake mushroom) is one of the most widely cultivated edible mushrooms and is highly valued for its medical applications (Chihara et al. 1969; Xu et al. 2011). In recent years, *L. edodes* has been primarily cultivated using mycelial blocks (sawdust medium cultivation) consisting of hardwood sawdust and nutritive materials, such as rice bran and wheat bran. Typically, the *L. edodes* mycelia inoculated on the surface (or inoculation hole) of the medium extends into the interior of the medium over a period of approximately 30 days. Following maturation of the mycelial block, fruiting bodies develop approximately 100 days after the inoculation. While the production technology is improving, *L. edodes* requires a longer cultivation time than other mushrooms, such as *Flammulina velutipes* and *Pleurotus ostreatus*, and the mechanism of mycelial growth and nutrient uptake during cultivation of mushrooms has not been elucidated (Balan et al. 2022).

*L. edodes* is a white-rot fungus that can break down wood cell wall components including cellulose, hemicellulose, pectin, and lignin using various extracellular enzymes. Crystalline cellulose is depolymerized by cellobiohydrolases belonging to glycoside hydrolase (GH) families 6 and 7 (CAZy database; http://www.cazy.org) (Drula. et al. 2022; Li et al. 2019; Uzcategui et al. 1991) and lytic polysaccharide monooxygenase (LPMO) categorized into auxiliary activity (AA) family 9 (Wymelenberg et al. 2002). Amorphous cellulose is mainly depolymerized by endoglucanases (GH families 5, 9, 12, 44, and 45) (Elisashvili et al. 2008; Henriksson et al. 1999). The constituents and composition of hemicellulose differ among plant species, for example, hardwoods have O-acetyl-4-methylglucuronoxylan as the major hemicellulose component. Hemicelluloses are distributed in a diverse and complex manner in plant cell walls, and correspondingly, mushrooms also have various GHs (Cai et al. 2017). In *L. edodes*, which prefers hardwoods, xylan degrading enzymes belonging to GH family 11 and xyloglucan degrading enzymes belonging to GH family 12 have been identified (Lee et al. 2005; Takeda et al. 2013). Degradation of pectin has been suggested to be important for the initial stages of wood decay (Zhang et al. 2016; Tanaka et al. 2019). In the case of fungi, most pectinases such as polygalacturonase are categorized into GH family 28. We have purified and characterized a pectin-degrading enzyme from *L. edodes* (*Le*PG28A) that has endotype polygalacturonase activity (Tanaka et al. 2020). White-rot fungi secrete enzymes that depolymerize lignin, such as heme peroxidase, laccase (Lcc), FAD-dependent oxidase, and dehydrogenase (Floudas et al. 2012; Levasseur et al. 2014). In *L. edodes* cultivation studies, secretions of Lcc and manganese peroxidases (MnP) have been reported (Boer et al. 2004; Forrester et al. 1990). A typical *L. edodes* mycelial block contains starch from rice bran and wheat bran, which is also enzymatically degraded by amylases (Zhao et al. 2000). The mycelia obtain nutrients from these enzymatic degradation products as a carbon source.

The cell wall of fungi such as *L. edodes* is constructed mainly from chitin and β-1,3/1,6-glucans (Shida et al. 1981). Therefore, fungi also produce GHs associated with the fungal cell wall polysaccharides, β-1,3-glucanases, β-1,6-glucanases, and chitinases. We previously analyzed the mRNA expression patterns of GHs in *L. edodes* mycelia, fruiting bodies at various growth stages, and post-harvest fruiting bodies (Sakamoto et al. 2017). A β-1,3-glucanase (GLU-1, GH family 128) (Sakamoto et al. 2011) and a β-hexosaminidase (*Le*Hex20A, GH family 20) (Konno et al. 2012) were especially upregulated in post-harvest shiitake mushrooms. A β-1,6-glucanase (*Le*Pus30A, GH family 30) (Konno and Sakamoto 2011) was highly expressed after harvest; however, the enzyme was also expressed in mycelia during liquid culture and in developing young fruiting bodies, suggesting that the enzyme is also involved in mycelial growth and fruiting body elongation. The expression of another β-hexosaminidase (*Le*HexB, GH family 20) (Konno et al. 2015) was higher in the mycelia and developing fruiting bodies than in the post-harvest fruiting bodies. Thus, *L. edodes* uses diverse autolysis enzymes in the course of morphological change.

As mentioned above, extracellular enzymes from *L. edodes* in mycelial blocks play an important role in sawdust medium cultivation. A spatial determination of the diversity and heterogeneity of enzyme distribution within mycelial blocks leads to the development of technologies and the solving of various problems in the edible mushroom cultivation. However, most studies on the production of these mycelial enzymes have been conducted in liquid culture or using fruiting bodies and little is known about cultivation in solid culture such as sawdust medium. Nagai et al. investigated the production of enzymes in *L. edodes* sawdust medium (Nagai et al. 2009; Nagai and Sato 2014). *L. edodes* mycelium was inoculated on sawdust medium in a petri dish, and enzymes were recovered and assayed based on the degree of mycelial growth on the surface of the medium. The study indicated that the secreted enzymes differed according to cultivation time and mycelial position, with Lcc activity in particular increasing at the early stage of cultivation at the mycelial tip. The mycelial tip region is the point of mycelial growth, suggesting that Lcc plays important roles for vegetative mycelial growth and colonization of the growth substrate. However, it is not clear whether the same enzyme secretion occurs in the mycelial blocks for fruiting body cultivation.

In this study, to obtain further insights into the differences in secreted enzymes depending on mycelial location, we investigated enzymatic activities in mycelial blocks after sawdust cultivation using bottles. Under conditions more similar to actual mycelial block cultivation than the above study (Nagai and Sato 2014), the mycelium was elongated longitudinally using bottle culture, divided into three sections with different mycelial ages (top, middle, and bottom parts of the mycelial block), and enzyme activities were analyzed for each individual section. We measured enzymatic degradation activities not only for wood cell wall polysaccharides, starch, and lignin, but also for fungal cell wall polysaccharides, such as β-1,3-glucanase, β-1,6-glucanase, and chitinase activities. Furthermore, we identified Lccs secreted in the mycelial tip region and analyzed the expression levels of their genes by real-time PCR.

## Materials and methods

### Reagents, fungal strain, and culture conditions

All reagents were purchased from Kanto Chemical Industry Co., Ltd. (Tokyo, Japan) or FUJIFILM Wako Pure Chemical Corporation (Osaka, Japan) unless otherwise noted. *L. edodes* strain H607 (Hokken Co., Ltd., Tochigi, Japan) was used in all experiments. The mycelia were maintained on potato dextrose agar (PDA; potato starch 4.0 g/L, dextrose 20 g/L and agar 15 g/L) medium. For sawdust medium cultivation, the medium was prepared with *Quercus serrata* (konara oak) sawdust / rice bran / wheat bran (9 / 0.5 / 0.5 by wet weight), the moisture content was adjusted to approximately 65%, and the prepared media were autoclaved at 121 °C for 30 min. The moisture content in the sawdust medium was measured using the moisture analyzer MOC63u (Shimadzu, Kyoto, Japan).

*L. edodes* mycelia from the PDA medium (7 mm diameter) were inoculated onto the center of the sawdust medium in a petri dish (90 mm diameter) and pre-cultured at 20 °C for 2 weeks. The 20 g of pre-cultured medium was inoculated on 220 g of sawdust medium (height of medium, 75 mm) in a mayonnaise bottle (width, 58 mm; height, 128 mm). The lids of bottles were made with a hole of approximately 1 cm in diameter and covered with autoclave tape. The sawdust medium bottles were incubated at 20 °C for 27 days; until the mycelial tips reached the bottom of the bottle. Culture experiments were conducted with 20 bottles.

**Fig. 1:**
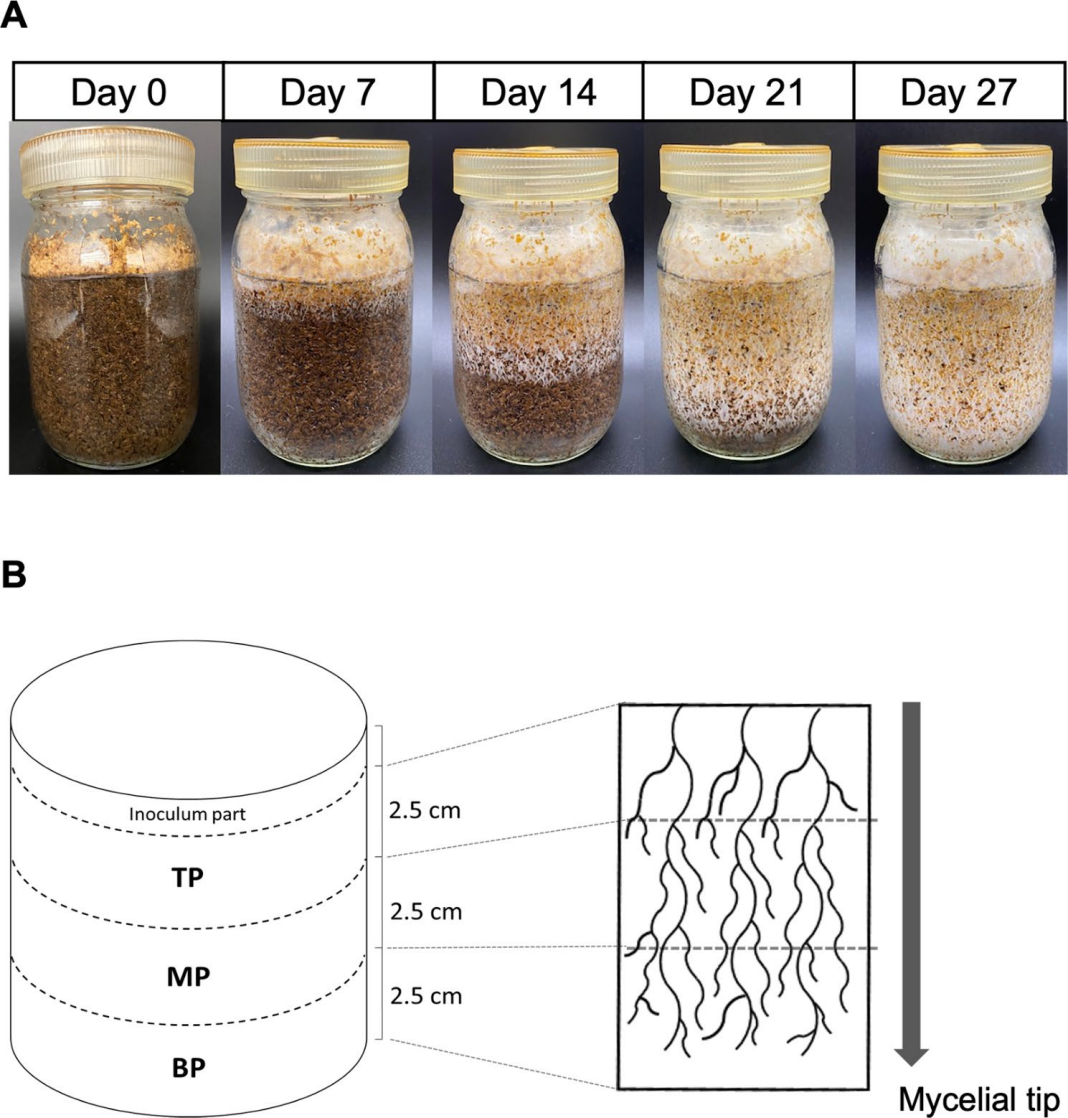
Growth process of mycelia on sawdust medium and recovery of the medium **(A)** Sawdust medium was placed in mayonnaise bottles to a height of 7.5 cm. The pre-cultured mycelium was inoculated on the top surface of the medium, and the mycelium elongated toward the bottom side. **(B)** The mycelium reached the bottom of the bottle at 27 days of incubation, and the medium was cut into three parts of 2.5 cm intervals and collected as the bottom part (BP), middle part (MP), and top part (TP).

At the end of incubation, each culture medium was removed from the bottle and collected in three parts (top, middle, and bottom parts), at 2.5 cm intervals from the bottom of the bottle (Fig. 1). Twenty culture bottles of sawdust medium were divided into the top, middle, and bottom parts, and the samples collected from each part were uniformly mixed and then stored at -25 °C (for analysis of enzyme) or at -80 °C (for analysis of mRNA).

### Enzyme extraction

Proteins were extracted from the medium for each of the three parts (10.0 g). The samples were crushed, suspended in 20 mL of 10 mM sodium phosphate buffer (pH 7.0), and incubated with rotation at 4 °C for 20 h. At the end of the incubation, the supernatant containing proteins was prepared by filtration with gauze and centrifugation (2,500 × *g*, 4 °C, 30 min). The supernatant was incubated with 5% (wt/vol) bentonite for 30 min, and the bentonite was removed by centrifugation (2,500 × *g*, 4 °C, 30 min). Ammonium sulfate was added to the supernatant until the concentration reached 70% saturation, and the resulting precipitate was dissolved in 2 mL of 50 mM sodium phosphate buffer (pH 7.0). The crude enzyme solution was desalted and concentrated using a VIVASPIN 10,000 NMWL filter (Cytiva, Tokyo, Japan). Protein concentration was measured by the Bradford method using the Bradford Dye Reagent (Takara Bio Inc., Shiga, Japan), with bovine serum albumin (BSA) (Merck Millipore, Darmstadt, Germany) as a standard. Protein concentration was monitored by absorbance at 595 nm.

### Enzyme assay

Laccase (Lcc) activity was measured using 1.25 mM 2,2-azino-bis (3-ethylbenzothiazoline-6-sulfonic acid) diammonium salt (ABTS, molecular extinction coefficient (ε) = 36,000 M^− 1^ cm^− 1^ at 420 nm) (Sigma-Aldrich Inc., St. Louis, MO, USA) in 50 mM sodium citrate buffer (pH 3.0) (Nakade et al. 2013). MnP activity was determined by monitoring the oxidation of guaiacol (3 mM) spectrophotometrically at 465 nm (ε = 12,000 M^− 1^ cm^− 1^) in 100 mM sodium acetate buffer (pH 5.0) with 20 mM MnSO_4_ and 0.1 mM H_2_O_2_ (Ergül et al. 2009).

Glycoside hydrolase activities were defined using 0.5% of substrates, cellulose powder (CP, cellulase assay for crystalline cellulose), sodium carboxymethyl cellulose (CMC, cellulase assay for amorphous cellulose), 4-O-methyl-D-glucurono-D-xylan (xylanase assay), polygalacturonic acid sodium salt (pectinase assay), soluble starch (amylase assay), laminarin (β-1,3-glucanase assay), and pustulan (β-1,6-glucanase assay), in 50 mM sodium acetate buffer (pH 5.0). A water-soluble cell wall polysaccharide (WSP) mixture from *L. edodes* fruiting bodies (Hokken) was obtained by hot-water extraction (120 °C, 20 min) as described previously (Konno et al. 2014), and WSP was used as the substrate for assay of fungal autolysis activity. The amount of reducing sugar generated in a reaction solution was measured by the Somogyi-Nelson method (Somogyi 1952).

Chitinase activity was measured by the amount of *N*-acetylglucosamine (GlcNAc) released from chitin using the Morgan-Elson assay according to the previously reported method (Keyhani and Roseman 1996; Konno et al. 2012). A water-insoluble cell wall polysaccharide (WIP) mixture from *L. edodes* fruiting bodies, obtained as a residue of the hot-water extraction described above, was also used as the substrate for the chitinase assays.

For all enzymatic assays, one unit (U) of enzyme activity was defined as the amount of enzyme that produces 1 µmol reaction products per minute.

### Laccase purification

Proteins with Lcc activity were purified from the protein extracts of the bottom-part of the cultured medium. Enzyme purification was performed at 4 °C unless otherwise stated. Samples (600 g) were crushed, suspended in 1,200 mL of 10 mM sodium phosphate buffer (pH 7.0), and incubated with rotation at 4 °C for 20 h. The crude enzyme solution was prepared as described above. Proteins were precipitated by ammonium sulfate (70% saturation), and the resulting precipitates were dissolved in 10 mM sodium phosphate buffer (pH 7.0) containing ammonium sulfate at 30% saturation. The supernatant was applied to a HiPrep™ Phenyl FF (high sub) 16/10 column (1.6 × 10 cm, Cytiva, Tokyo, Japan) equilibrated with 10 mM sodium phosphate buffer containing 30% ammonium sulfate. After 90 mL of the same buffer was passed through the column, a linear gradient of ammonium sulfate (30 − 0%) was applied at a flow rate of 1.5 mL/min to elute the adsorbed protein. Laccase activity was measured using ABTS, and the active fractions were collected. The sample was concentrated using a 10 kDa molecular weight cut-off membrane (Vivaspin™ 10 K, Cytiva), and the solvent was replaced with 10 mM sodium phosphate buffer. The resulting enzyme solution was applied to a TOYOPEARL SuperQ-650 M column (2.2 × 9.5 cm, TOSOH, Tokyo, Japan) equilibrated with 10 mM sodium phosphate buffer. The adsorbed proteins were eluted with a linear gradient of NaCl (0-0.5 M) at a flow rate of 1.5 mL/min. The active fractions were concentrated and desalted by ultrafiltration (Vivaspin™ 10 K, Cytiva). The concentrated enzyme solution was applied to a TSK gel G3000SW_XL_ column (7.8 mm I.D. × 30 cm, TOSOH) equilibrated with 25 mM sodium phosphate buffer containing 0.3 M NaCl. The proteins were eluted using the same eluent at a flow rate of 0.5 mL/min, and absorbance was monitored at 280 nm. The obtained active fractions were analyzed by SDS-PAGE (12% [w/v] polyacrylamide), and proteins were identified by LC-MS/MS as described previously using a Triple TOF 5600+ mass spectrometer (AB Sciex, Framingham, MA, USA) (Tanaka et al. 2019). The Peptide sequencing was performed using PEAKS Studio v10 (Bioinformatics Solutions Inc., Waterloo, Canada) with the protein database of *Lentinula edodes* (Accession: PRJDB4944, NCBI) (Sakamoto et al. 2017).

### Analysis of mRNA levels of laccase genes by real-time PCR

Total RNA from the medium for each of the three parts using an easy-spin™ total RNA Extraction Kit (iNtRON biotechnology, Jungwon-Gu, Korea), and cDNA was synthesized from total RNA extracted using a PrimeScript™ RT Reagent Kit (Takara Bio Inc.) according to the manufacturer’s protocols. Real-time PCR was performed with THUNDERBIRD™ SYBR® qPCR MIX (TOYOBO, Osaka, Japan) on a LightCycler®96 Real-Time PCR System (Roche, Basel, Switzerland). To analyze the level of transcription of laccase (Lcc1, Lcc5, Lcc6 and Lcc13) encoding genes, *lcc1*-specific primers (5’-ACGTCGCCGCCGTTAAT-3’ and 5’-GCATCATAAGTTGGGCAAAGTTG-3’), *lcc5*-specific primers (5’-CAGCGGTGCGGAAACG-3’ and 5’-TGGTGGGCAAGGAGTAAACAG-3’), *lcc6*-specific primers (5’-CATCTGGAGGCTGGATTTGC-3’ and 5’-TTGTCGGATTAGCGGAAGCA-3’) and *lcc13*-specific primers (5’-CCAGGAACTTGAACCAAAACG-3’ and 5’-GGCGCACCTTTGTATCGTAAG-3’) were used (Sakamoto et al. 2015). The real-time PCR data were normalized against the glyceraldehyde-3-phosphate dehydrogenase gene (*gpd*) expression (Hirano et al. 1999) detected with *gpd*-specific primers (5’-TGTTATCTCCAATGCTTCTTGCA-3’ and 5’-CCGAATTTGTCGTGGATAACCT-3’). The expression patterns were analyzed by ΔΔC_T_ method (Livak and Schmittgen 2001) with three replicates, and the expression level of the top part of the cultured medium was used as a calibrator.

## Results

### Enzymatic activities in the sawdust medium

The mycelia inoculated on the top surface of the medium grew downward with the duration of culture (Fig. 1). The growth rate of mycelia was similar among the 20 culture bottles tested, and the cultivation was terminated when the tip of the mycelium reached the bottom of the bottle after 27 days of incubation. The pH of the medium at the initial incubation was 4.9, but the pH of the recovered beds decreased to 3.9, 4.2, and 4.4 for the top, middle, and bottom, respectively, showing a trend toward lower pH in the upper part. The result indicates that mycelial maturation is more advanced in the top part than in the bottom part. Protein contents in the prepared extracts from the top, middle, and bottom parts were 0.085, 0.090, 0.072 mg/g (per wet weight of the medium), respectively.

**Fig. 2:**
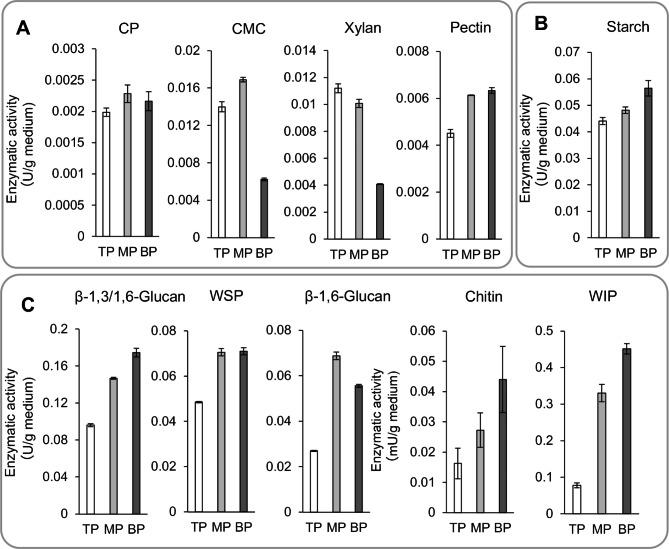
Carbohydrate hydrolase activities in the sawdust medium cultivated for 27 days Activities were measured using various substrates: **(A)** plant cell wall polysaccharides such as cellulose powder (CP), sodium carboxymethyl cellulose (CMC), 4-O-methyl-D-glucurono-D-xylan (xylan), polygalacturonic acid sodium salt (pectin), **(B)** nutritive materials such as soluble starch (starch), and **(C)** fungal cell wall polysaccharides such as laminarin (β-1,3/1,6-glucan), pustulan (β-1,6-glucan), chitin, hot-water soluble and insoluble cell wall polysaccharides mixture from *L. edodes* (WSP and WIP). TP, top part; MP, middle part; BP, bottom part. Values are means ± SD of three independent experiments

Enzymatic degradation activities were measured using wood cell wall polysaccharides (Fig. 2A), starch (Fig. 2B), and fungal cell wall polysaccharides (Fig. 2C) as substrates. Among activities for wood cell wall polysaccharides, pectinase activity in the bottom part was 1.4-fold higher than that in the top part. Starch degradation (amylase) activity was also higher (1.3-fold) in the bottom part than in the top part. Therefore, it was suggested that pectinase and amylase are predominantly secreted near the tip of the mycelium.

In contrast, the hydrolase activities for CMC and xylan were higher in the top and middle parts of the medium. The CMC and xylan degradation activities in the top part were 2.2 and 2.7-fold higher than those in the bottom part, respectively. The results indicate that the enzymes involved with hemicellulose degradation such as endoglucanase and xylanase are secreted from the maturing mycelium, not from the mycelial tips. Cellulase activity for CP was low in all parts of the 27-day cultivated medium, suggesting that crystalline cellulose degradation occurred after hemicellulose degradation.

Enzymatic degradation activities for fungal cell wall polysaccharides were measured using laminarin (β-1,3/1,6-glucan), pustulan (β-1,6-glucan), chitin, and the water-soluble and -insoluble polysaccharides extracted from *L. edodes* fruiting bodies, WSP and WIP (Fig. 2C). The laminarin, pustulan and chitin degradation activities in the bottom part were 1.8, 2.1, and 2.7-fold higher than those in the top part, respectively. The degradation activities for WSP (containing β-1,3/1,6-glucans) and WIP (containing chitin) were also higher in the bottom and middle parts. The results indicate that degradation enzymes associated with the fungal cell wall such as β-1,3-glucanases, β-1,6-glucanases, and chitinases are highly produced in the mycelial tip region, i.e., the point of mycelial growth. Furthermore, the highest degradation activities of laminarin and chitin were observed in the lower part, while the highest degradation activity of pustulan was observed in the middle part, suggesting that β-1,6-glucanase is produced after β-1,3-glucanase and chitinase.

**Fig. 3:**
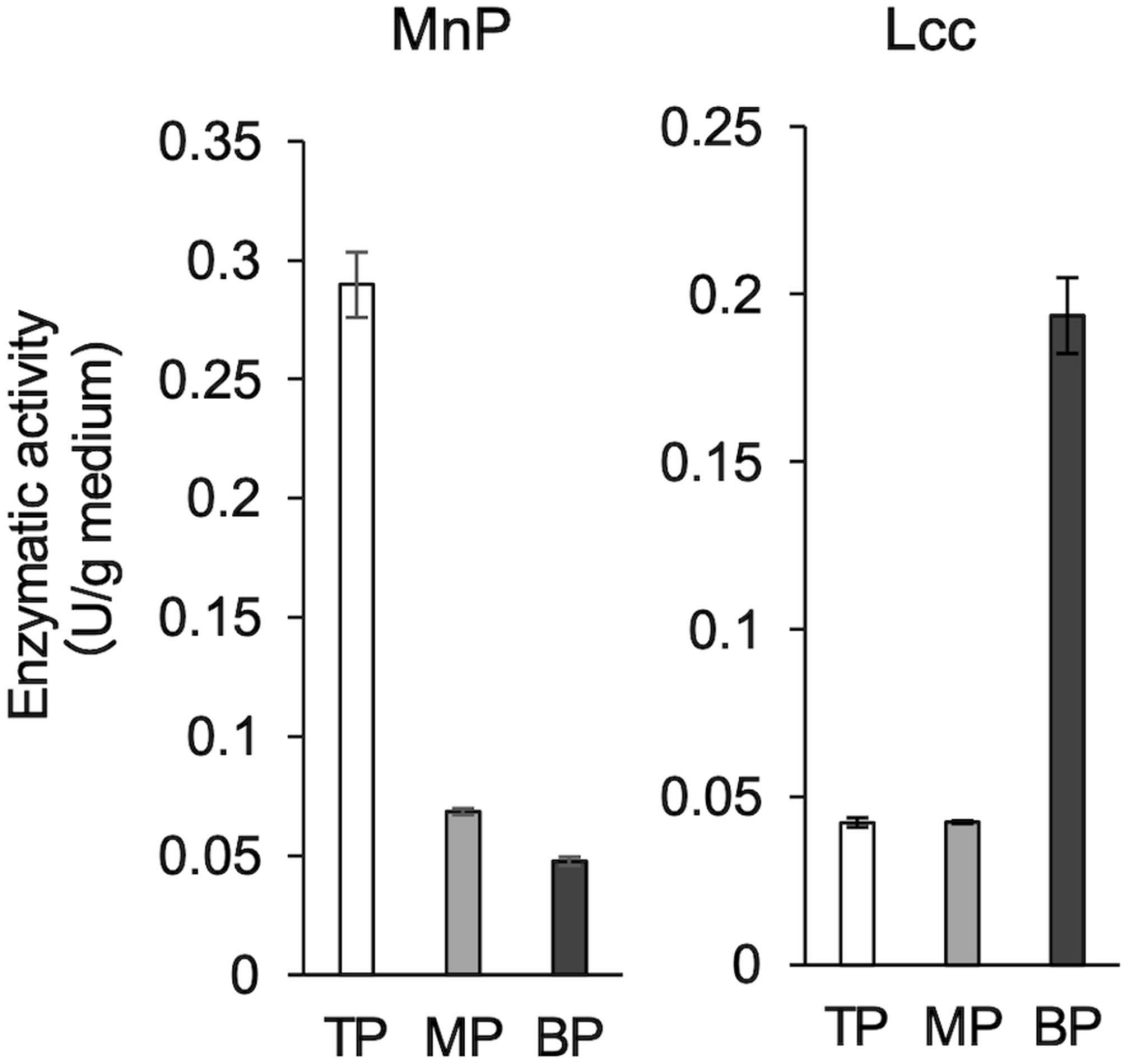
Manganese peroxidase (MnP) and laccase (Lcc) activities in the sawdust medium cultivated for 27 days. MnP activity was measured using guaiacol as the substrate, while Lcc activity was measured using ABTS as the substrate. TP, top part; MP, middle part; BP, bottom part. Values are means ± SD of three independent experiments

MnP and Lcc activities were determined using guaiacol and ABTS as substrates, respectively (Fig. 3). MnP activity was the highest in the top part, 6.1-fold higher than in the bottom part (Fig. 3). On the other hand, Lcc activity was obviously higher in the bottom part compared to that of the other parts (approximately 4.6-fold; Fig. 3), indicating that Lccs are secreted predominantly at the mycelial tip region.

### Laccases in the bottom part of the medium

**Fig. 4:**
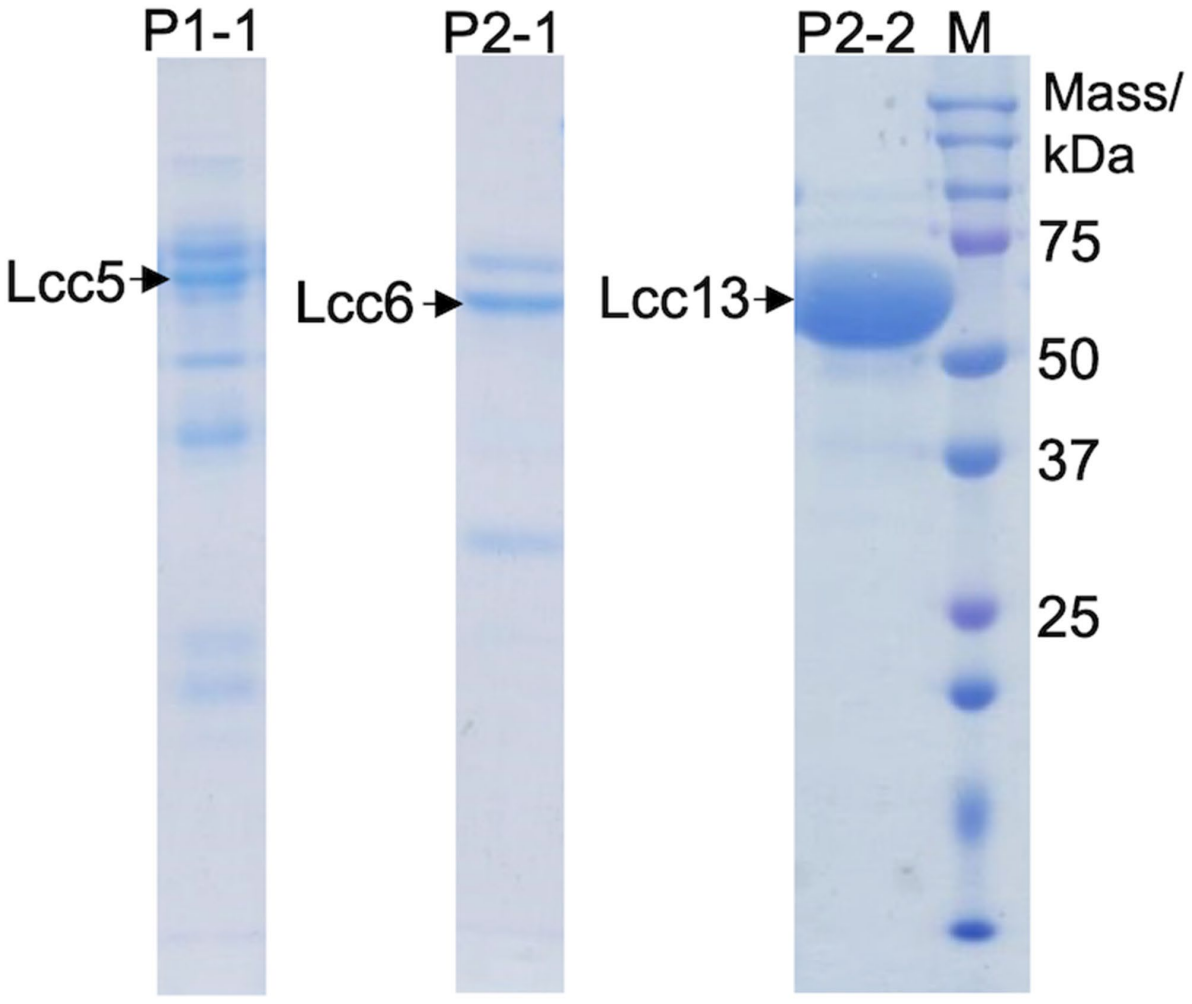
SDS-PAGE pattern of the partially purified Lccs. The proteins with Lcc activity were purified from extracts of the bottom part of the cultured sawdust medium. The obtained active fractions, P1-1, P2-1, and P2-2, were applied to SDS-PAGE. The obtained bands were applied to LC-MS/MS analysis, and three Lccs, Lcc5 (molecular mass of 68 kDa, from P1-1), Lcc6 (55 kDa, from P2-1), and Lcc13 (61 kDa, from P2-2), were identified

As Lccs have important roles for vegetative mycelial growth, we fractionated enzymes with Lcc activity from the bottom part of the medium. The crude enzymes were sequentially subjected to fractionation by hydrophobic, anion-exchange and size-exclusion column chromatography, and the Lcc purification was performed based on the enzymatic activity for ABTS (Additional file 1: Figure S1). As the result of the column chromatograpies, three partially purified fractions with Lcc activity, P1-1 (1.1 U/mg), P2-1 (11.4 U/mg) and P2-2 (4.9 U/mg), were obtained.These fractions were applied to SDS-PAGE (Fig. 4), and the distinctive bands on the active fractions were identified by LC-MS/MS. From the analysis, three laccase candidates, Lcc5 (from P1-1; Accession No., XP_046088042.1; coverage, 10%), Lcc6 (from P2-1; Accession No., BAJ12090.1; coverage, 11%) and Lcc13 (from P2-2; Accession No., BAT31655.1; coverage, 26%), were identified (Additional file 1: Figure S2).

**Fig. 5:**
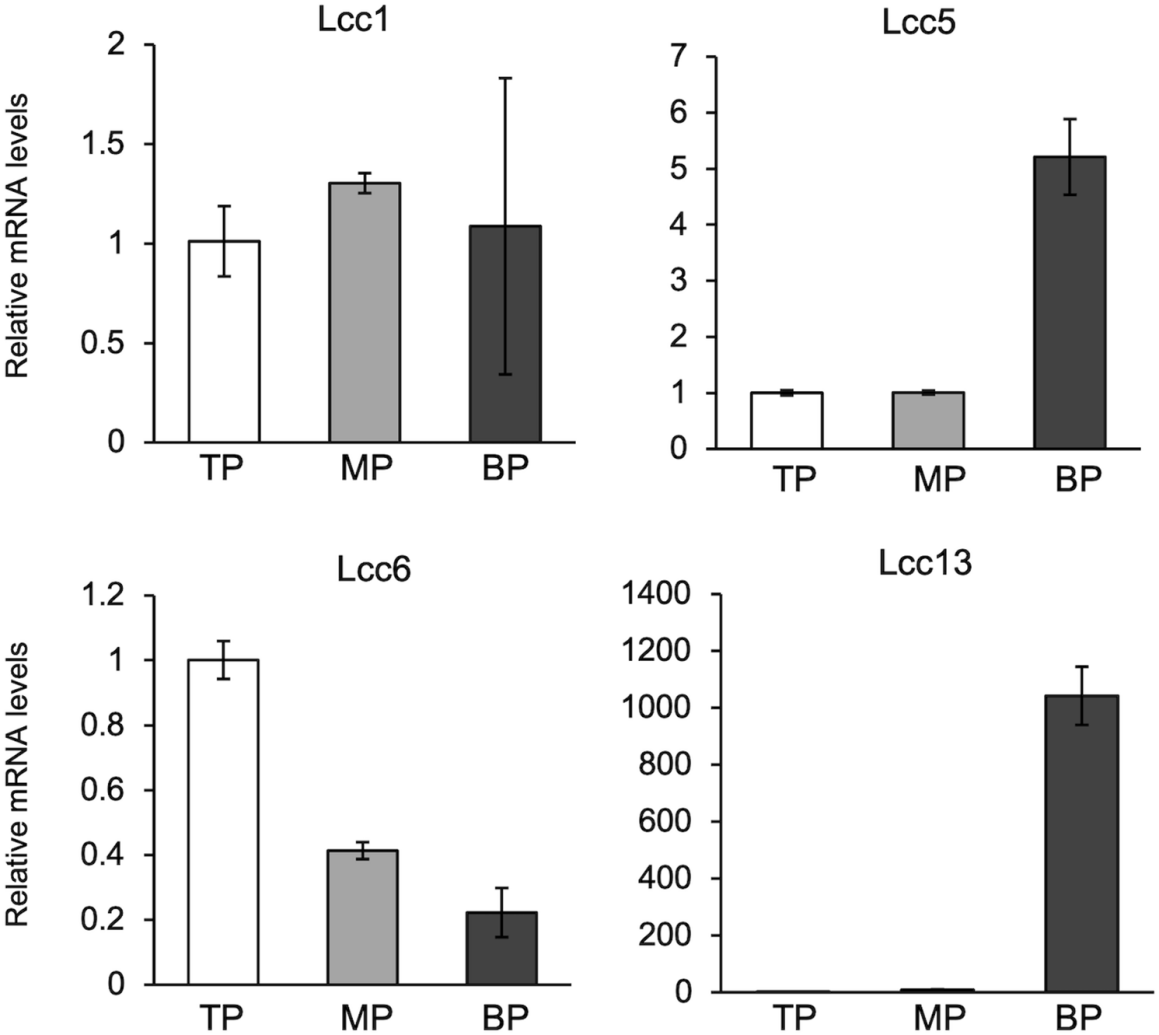
Analysis of the transcription level of Lcc1, Lcc5, Lcc6, Lcc13 encoding genes using real-time PCR. TP, top part; MP, middle part; BP, bottom part. Quantities of mRNAs were relative to the level in TP. All data points are means ± SD (n = 3)

The expression patterns of *lcc1* (for comparison), *lcc5*, *lcc6* and *lcc13* in the parts of the medium were analyzed by real-time PCR (Fig. 5). The expressions of *lcc5* and *lcc13* were more highly in the bottom part than in the top part. In particular, the *lcc13* expression was 1,042-fold higher in the bottom part compared with the level in the top part, suggesting Lcc13 is mainly secreted at the mycelial tip region. In contrast, *lcc6* was transcribed in the top part, but at a lower level in the bottom part. *lcc1*, a well characterized laccase gene in *L. edodes*, showed no significant expression differences among the midium sites.

## Discussion

Sawdust medium cultivation of *L. edodes* is divided into four main stages: (1) mycelial spread and nutrient uptake, (2) mycelial maturation (surface browning), (3) primordial formation, and (4) fruiting body growth. The *L. edodes* mycelium produces unique enzymes at each of these growth stages. In this study, mycelial characteristics during the first step of the cultivation cycle, i.e., mycelial spread and vegetative growth, was investigated based on the differences in enzyme activities among different parts of the mycelium. Most of the enzymes produced by mycelia are located around the mycelia and enzymatically degrade the plant biomass in the sawdust medium. The low-molecular-weight products are incorporated into the mycelium as nutrients. If the mycelium does not spread normally in the sawdust medium during this stage, energy intake is insufficient, resulting in poor subsequent fruiting body formation. Therefore, the ability of the mycelium to expand deep into the plant cell wall during this early stage of culture has a significant influence on fruiting body yield.

In the mycelial tip region (in the bottom part of the sawdust medium), amylase, pectinase, fungal cell wall degradation enzymes (β-1,3-glucanase, β-1,6-glucanase, and chitinase), and Lcc were highly secreted. On the other hand, lignocellulose degradation enzymes, such as endoglucanase (CMCase), xylanase, and MnP were highly secreted in the top part, where the relatively mature mycelium was present. The results indicate that the initial sawdust degradation occurs after mycelial colonization and the enzymes produced from the tip region act in the first step of *L. edodes* colonization. Similar trends have been reported in the mycelial block of *Pleurotus ostreatus*, e.g., Lcc is produced during vegetative stage, followed by MuP (Bánfi et al. 2015).

Amylase is known to degrade the starch in nutritive materials such as rice bran and wheat bran, indicating that these starch components are utilized prior to other polysaccharides in the medium. The same phenomenon was observed in a previous study using *L. edodes* mycelium (Leatham 1985; Nagai and Sato 2014). Pectin is a major component of the primary wall and the middle lamella of the plant cell wall, and is likely important for cellular adhesion (Siedlecka et al. 2008). In brown rot fungi, enzymatic degradation of pectin in wood is involved in the initial stage of wood decay, and it has been suggested that pectinolytic enzymes are secreted from the mycelial tip region (Presley et al. 2017; Tanaka et al. 2023; Zhang et al. 2016). Specifically, this process appears to be important for the initial disruption of the wood cell wall, facilitating fungal access. The results of our study indicated that *L. edode*s, a white rot fungus, also secretes pectinase from near the tip of the mycelium at the early stage of growth to promote mycelial colonization.

In the bottom part of the sawdust medium, high β-1,3-glucanase, β-1,6-glucanase, and chitinase activities were detected. These fungal cell wall degradation enzymes were considered to contribute to mycelial growth and development, as the mycelial tip region is the point of mycelial growth with active cell division. The contribution of autolysis enzymes to mycelial growth with the relevant synthesizing enzymes is supported by previous studies on *L. edodes* enzymes (Konno and Sakamoto 2011; Konno et al. 2015). Furthermore, Leatham et al. reported that β-1,3-glucanase activity was correlated with the nutrient growth rate of mycelia, suggesting that its activity is important for colonization of the mycelial block (Leatham et al. 1985). The involvement of β-1,3-glucanase in mycelial growth has been mentioned in previous studies. In addition to that, the present study is the first to show that β-1,6-glucanase and chitinase also contribute to mycelial growth.

Lcc activity was also significantly higher in the mycelial tip region. The 12 Lcc genes are conserved in the *L. edodes* genome and are thought to have versatile functions, such as lignin degradation, mycelial cell wall synthesis, and fruiting body coloration (Sakamoto et al. 2015). Nagai et al. reported that the addition of Lcc1 to the culture medium increased the substrate sensitivity of cellulase and xylanase, suggesting that laccases can partially degrade lignin in sawdust and facilitate the degradation of lignocellulose (Nagai and Sato 2014). The authors have reported that the mycelial cell wall become thinner in a Lcc1 downregulated strain, which also contributes to mycelial cell wall morphogenesis (Sakamoto et al. 2018). Several laccases are secreted in mycelial blocks, and Lcc6 has been purified and characterized (Nagai et al. 2009). Sakamoto et al. reported that Lcc5 is secreted near the mycelial tip (Sakamoto et al. 2015). Although Lcc1 was not obtained from the bottom part of the sawdust medium in this study, we showed that Lcc13 is also secreted in the mycelial block, in addition to Lcc5 and Lcc6. The expression of Lcc13 gene was extremely high at the mycelial tip region compared to other regions, suggesting that optimal secretion of Lcc13 is essential for mycelial colonization in the sawdust medium. Cultivation of shiitake mushrooms is more time consuming than other mushroom species, and the mycelial condition can only be evaluated visually. We believe that the detection of Lcc13 provides a valid preliminary assessment of the mycelium blocks. In order to clarify the role of Lcc13 and other isozymes in the mycelial colonization, more detailed characterization of the laccases produced from the mycelial tip are in progress.

## Electronic supplementary material

Below is the link to the electronic supplementary material.

Additional file 1: Figure S1. Laccase purification from the bottom part of the medium. The Lcc purification was performed based on the enzymatic activity for ABTS. (A) Hydrophobic chromatogram of the crude enzyme on HiPrep™ Phenyl FF (high sub) 16/10 column. Arrows show recovered fractions, P1(Fr. 39–47) and P2 (48–57). (B) Anion exchange chromatogram of P1 on TOYOPEARL SuperQ-650 M column. An active fraction, Fr. 40, was recovered. (C) Anion exchange chromatogram of P2 on TOYOPEARL SuperQ-650 M column. Active fractions, Fr. 30–33, were recovered. The obtained active fractions were further applied to size exclusion chromatography, and the 3 partially purified fractions, P1-1 (from P1), P2-1 and P2-2 (from P2), were obtained.
Figure S2. Coverage maps of peptide fragments of Lcc5, Lcc6 and Lcc13. The SDS-PAGE bands were subjected to trypsin digestion and LC-MS/MS analysis and compared with the protein database of *Lentinula edodes* (Accession, PRJDB4944) (Sakamoto et al. 2017). *De novo* peptide sequencing was performed using PEAKS Studio v10. Homology searches were performed with BLAST (NCBI). Blue lines indicate digestible fragments analyzed with total sequence coverages of Lcc5 (10%), Lcc6 (11%) and Lcc13 (26%).

## Data Availability

The datasets used and/or analyzed during the current study are available from the corresponding author on reasonable request.

## Abbreviations

*L. edodes*: *Lentinula edodes*
GH: glycoside hydrolase
Lcc: laccase
MnP: manganese peroxidase
CMC: carboxymethyl cellulose
CP: cellulose powder
WSP: water-soluble cell wall polysaccharide mixture from *L. edodes* fruiting bodies
WIP: water-insoluble cell wall polysaccharide mixture from *L. edodes* fruiting bodies
ABTS: 2,2-azino-bis (3-ethylbenzo-thiazoline-6-sulfolic acid) diammonium salt

## References

[CR1] Balan V, Zhu W, Krishnamoorthy H, Benhaddou D, Mowrer J, Husain H, Eskandari A (2022). Challenges and opportunities in producing high-quality edible mushrooms from lignocellulosic biomass in a small scale. Appl Microbiol Biotechnol.

[CR2] Bánfi R, Pohner Z, Kovács J, Luzics S, Nagy A, Dudás M, Tanos P, Márialigeti K, Vajna B (2015). Characterisation of the large-scale production process of oyster mushroom (*Pleurotus ostreatus*) with the analysis of succession and spatial heterogeneity of lignocellulolytic enzyme activities. Fungal Biol.

[CR3] Boer CG, Obici L, de Souza CG, Peralta RM (2004). Decolorization of synthetic dyes by solid state cultures of *Lentinula* (*Lentinus*) *edodes* producing manganese peroxidase as the main ligninolytic enzyme. Bioresour Technol.

[CR4] Cai Y, Gong Y, Liu W, Hu Y, Chen L, Yan L, Zhou Y, Bian Y (2017). Comparative secretomic analysis of lignocellulose degradation by *Lentinula edodes* grown on microcrystalline cellulose, lignosulfonate and glucose. J Proteom.

[CR5] Chihara G, Maeda Y, Hamuro J, Sasaki T, Fukuoka F (1969). Inhibition of mouse sarcoma 180 by polysaccharides from *Lentinus edodes* (Berk.) Sing. Nature.

[CR6] Drula E, Garron ML, Dogan S, Lombard V, Henrissat B, Terrapon N (2022). The carbohydrate-active enzyme database: functions and literature. Nucleic Acids Res.

[CR7] Elisashvili V, Penninckx M, Kachlishvili E, Tsiklauri N, Metreveli E, Kharziani T, Kvesitadze G (2008). *Lentinus edodes* and *Pleurotus* species lignocellulolytic enzymes activity in submerged and solid-state fermentation of lignocellulosic wastes of different composition. Bioresour Technol.

[CR8] Ergül FE, Sargın S, Öngen G, Sukan FV (2009). Dephenolisation of olive mill wastewater using adapted *Trametes versicolor*. Int Biodeterior Biodegradation.

[CR9] Floudas D, Binder M, Riley R, Barry K, Blanchette RA, Henrissat B, Martínez AT, Otillar R, Spatafora JW, Yadav JS, Aerts A, Benoit I, Boyd A, Carlson A, Copeland A, Coutinho PM, de Vries RP, Ferreira P, Findley K, Foster B, Gaskell J, Glotzer D, Górecki P, Heitman J, Hesse C, Hori C, Igarashi K, Jurgens JA, Kallen N, Kersten P, Kohler A, Kües U, Kumar TK, Kuo A, LaButti K, Larrondo LF, Lindquist E, Ling A, Lombard V, Lucas S, Lundell T, Martin R, McLaughlin DJ, Morgenstern I, Morin E, Murat C, Nagy LG, Nolan M, Ohm RA, Patyshakuliyeva A, Rokas A, Ruiz-Dueñas FJ, Sabat G, Salamov A, Samejima M, Schmutz J, Slot JC, St John F, Stenlid J, Sun H, Sun S, Syed K, Tsang A, Wiebenga A, Young D, Pisabarro A, Eastwood DC, Martin F, Cullen D, Grigoriev IV, Hibbett DS (2012). The paleozoic origin of enzymatic lignin decomposition reconstructed from 31 fungal genomes. Science.

[CR10] Forrester IT, Grabski AC, Mishra C, Kelley BD, Strickland WN, Leatham GF, Burgess RR (1990). Characteristics and N-terminal amino acid sequence of a manganese peroxidase purified from *Lentinula edodes* cultures grown on a commercial wood substrate. Appl Microbiol Biotechnol.

[CR11] Henriksson G, Nutt A, Henriksson H, Pettersson B, Ståhlberg J, Johansson G, Pettersson G (1999). Endoglucanase 28 (Cel12A), a new *Phanerochaete chrysosporium* cellulase. Eur J Biochem.

[CR12] Hirano T, Sato T, Okawa K, Kanda K, Yaegashi K, Enei H (1999). Isolation and characterization of the glyceraldehyde-3-phosphate dehydrogenase gene of *Lentinus edodes*. Biosci Biotechnol Biochem.

[CR13] Keyhani NO, Roseman S (1996). The chitin catabolic cascade in the marine bacterium *Vibrio furnissii*. Molecular cloning, isolation, and characterization of a periplasmic β-N-acetylglucosaminidase. J Biol Chem.

[CR16] Konno N, Sakamoto Y (2011). An endo-β-1,6-glucanase involved in *Lentinula edodes* fruiting body autolysis. Appl Microbiol Biotechnol.

[CR15] Konno N, Takahashi H, Nakajima M, Takeda T, Sakamoto Y (2012). Characterization of β-*N*-acetylhexosaminidase (LeHex20A), a member of glycoside hydrolase family 20, from *Lentinula edodes* (shiitake mushroom). AMB Express.

[CR14] Konno N, Nakade K, Nishitani Y, Mizuno M, Sakamoto Y (2014). Lentinan degradation in the *Lentinula edodes* fruiting body during postharvest preservation is reduced by downregulation of the exo-β-1,3-glucanase EXG2. J Agric Food Chem.

[CR17] Konno N, Obara A, Sakamoto Y (2015). Molecular cloning, characterization and expression analysis of a β-*N*-acetylhexosaminidase (LeHex20B) from the shiitake mushroom, *Lentinula edodes*. J Wood Sci.

[CR18] Lee CC, Wong DW, Robertson GH (2005). Cloning and characterization of the xyn11A gene from *Lentinula edodes*. Protein J.

[CR19] Levasseur A, Lomascolo A, Chabrol O, Ruiz-Dueñas FJ, Boukhris-Uzan E, Piumi F, Kües U, Ram AF, Murat C, Haon M, Benoit I, Arfi Y, Chevret D, Drula E, Kwon MJ, Gouret P, Lesage-Meessen L, Lombard V, Mariette J, Noirot C, Park J, Patyshakuliyeva A, Sigoillot JC, Wiebenga A, Wösten HA, Martin F, Coutinho PM, de Vries RP, Martínez AT, Klopp C, Pontarotti P, Henrissat B, Record E (2014). The genome of the white-rot fungus *pycnoporus cinnabarinus*: a basidiomycete model with a versatile arsenal for lignocellulosic biomass breakdown. BMC Genomics.

[CR20] Li L, Qu M, Liu C, Pan K, Xu L, OuYang K, Song X, Li Y, Zhao X (2019). Expression of a recombinant *Lentinula edodes* cellobiohydrolase by *Pichia pastoris* and its effects on in vitro ruminal fermentation of agricultural straws. Int J Biol Macromol.

[CR21] Livak KJ, Schmittgen TD (2001). Analysis of relative gene expression data using real-time quantitative PCR and the 2(-Delta Delta C(T)) method. Methods.

[CR23] Nagai M, Sato T (2014). Production of plant cell wall-degrading enzymes in *Lentinula edod*es and the important role of laccase in early stages of solid-state cultivation. Mushroom Sci Biotechnol.

[CR22] Nagai M, Sakamoto Y, Nakade K, Sato T (2009). Purification of a novel extracellular laccase from solid-state culture of the edible mushroom *Lentinula edodes*. Mycoscience.

[CR24] Nakade K, Nakagawa Y, Yano A, Konno N, Sato T, Sakamoto Y (2013). Effective induction of *pblac1* laccase by copper ion in *Polyporus brumalis* ibrc05015. Fungal Biol.

[CR25] Presley GN, Schilling JS (2017). Distinct growth and secretome strategies for two taxonomically divergent brown rot fungi. Appl Environ Microbiol.

[CR26] Sakamoto Y, Nakade K, Konno N (2011). Endo-β-1,3-glucanase GLU1, from the fruiting body of *Lentinula edodes*, belongs to a new glycoside hydrolase family. Appl Environ Microbiol.

[CR29] Sakamoto Y, Nakade K, Yoshida K, Natsume S, Miyazaki K, Sato S, van Peer AF, Konno N (2015). Grouping of multicopper oxidases in *Lentinula edodes* by sequence similarities and expression patterns. AMB Express.

[CR27] Sakamoto Y, Nakade K, Sato S, Yoshida K, Miyazaki K, Natsume S, Konno N (2017). *Lentinula edodes* genome survey and postharvest transcriptome analysis. Appl Environ Microbiol.

[CR28] Sakamoto Y, Nakade K, Sato S, Yoshimi A, Sasaki K, Konno N, Abe K (2018). Cell wall structure of secreted laccase-silenced strain in *Lentinula edodes*. Fungal Biol.

[CR30] Shida M, Ushioda Y, Nakajima T, Matsuda K (1981). Structure of the alkali-insoluble skeletal glucan of *Lentinus edodes*. J Biochem.

[CR31] Siedlecka A, Wiklund S, Péronne MA, Micheli F, Lesniewska J, Sethson I, Edlund U, Richard L, Sundberg B, Mellerowicz EJ (2008). Pectin methyl esterase inhibits intrusive and symplastic cell growth in developing wood cells of Populus. Plant Physiol.

[CR32] Somogyi M (1952). Notes on sugar determination. J Biol Chem.

[CR33] Takeda T, Nakano Y, Takahashi M, Sakamoto Y, Konno N (2013). Polysaccharide-inducible endoglucanases from *Lentinula edodes* exhibit a preferential hydrolysis of 1,3 – 1,4-β-glucan and xyloglucan. J Agric Food Chem.

[CR35] Tanaka Y, Suzuki T, Nakamura L, Nakamura M, Ebihara S, Kurokura T, Iigo M, Dohra H, Habu N, Konno N (2019). A GH family 28 endo-polygalacturonase from the brown-rot fungus *fomitopsis palustris*: purification, gene cloning, enzymatic characterization and effects of oxalate. Int J Biol Macromol.

[CR34] Tanaka Y, Hirama K, Suzuki T, Habu N, Konno N (2020). Purification and characterization of endo-polygalacturonase from white-rot fungus *Lentinula edodes*. Mushroom Sci Biotechnol.

[CR36] Tanaka Y, Nezu I, Aiso H, Fujie T, Konno N, Suzuki T, Ishiguri F, Habu N (2023). Pectin decomposition at the early stage of brown-rot decay by *Fomitopsis palustris*. Biosci Biotechnol Biochem.

[CR37] Uzcategui E, Ruiz A, Montesino R, Johansson G, Pettersson G (1991). The 1,4-β-D-glucan cellobiohydrolases from *Phanerochaete chrysosporium*. I. A system of synergistically acting enzymes homologous to *Trichoderma reesei*. J Biotechnol.

[CR38] Wymelenberg AV, Denman S, Dietrich D, Bassett J, Yu X, Atalla R, Predki P, Rudsander U, Teeri TT, Cullen D (2002). Transcript analysis of genes encoding a family 61 endoglucanase and a putative membrane-anchored family 9 glycosyl hydrolase from *Phanerochaete chrysosporium*. Appl Environ Microbiol.

[CR39] Xu X, Pan C, Zhang L, Ashida H (2011). Immunomodulatory beta-glucan from *Lentinus edodes* activates mitogen-activated protein kinases and nuclear factor-kappab in murine RAW 264.7 macrophages. J Biol Chem.

[CR40] Zhang J, Presley GN, Hammel KE, Ryu JS, Menke JR, Figueroa M, Hu D, Orr G, Schilling JS (2016). Localizing gene regulation reveals a staggered wood decay mechanism for the brown rot fungus *postia placenta*. Proc Natl Acad Sci U S A.

[CR41] Zhao J, Chen YH, Kwan HS (2000). Molecular cloning, characterization, and differential expression of a glucoamylase gene from the basidiomycetous fungus *Lentinula edodes*. Appl Environ Microbiol.

[CR42] Gary F., Leatham (1985) Extracellular Enzymes Produced by the Cultivated Mushroom Lentinus edodes during Degradation of a Lignocellulosic Medium. Appl Environ Microbiol 50(4) 859-867. 10.1128/aem.50.4.859-867.1985 10.1128/aem.50.4.859-867.1985 PMC291760 16346918

