# Supplementary material for: Extracellular enzymes secreted in the mycelial block of Lentinula edodes during hyphal growth
Source: AMB Express. 2023 Apr 25;13:36. doi: 10.1186/s13568-023-01547-6 (PMC10130320; doi:10.1186/s13568-023-01547-6)
Supplement: Supplementary file 1 — Additional file 1: Figure S1. Laccase purification from the bottom part of the medium. The Lcc purification was performed based on the enzymatic activity for ABTS. (A) Hydrophobic chromatogram of the crude enzyme on HiPrep™ Phenyl FF (high sub) 16/10 column. Arrows show recovered fractions, P1(Fr. 39–47) and P2 (48–57). (B) Anion exchange chromatogram of P1 on TOYOPEARL SuperQ-650 M column. An active fraction, Fr. 40, was recovered. (C) Anion exchange chromatogram of P2 on TOYOPEARL SuperQ-650 M column. Active fractions, Fr. 30–33, were recovered. The obtained active fractions were further applied to size exclusion chromatography, and the 3 partially purified fractions, P1-1 (from P1), P2-1 and P2-2 (from P2), were obtained. Figure S2. Coverage maps of peptide fragments of Lcc5, Lcc6 and Lcc13. The SDS-PAGE bands were subjected to trypsin digestion and LC-MS/MS analysis and compared with the protein database of Lentinula edodes (Accession, PRJDB4944) (Sakamoto et al. 2017). De novo peptide sequencing was performed using PEAKS Studio v10. Homology searches were performed with BLAST (NCBI). Blue lines indicate digestible fragments analyzed with total sequence coverages of Lcc5 (10%), Lcc6 (11%) and Lcc13 (26%). [file 13568_2023_1547_MOESM1_ESM.pdf]

Additional file 1

A

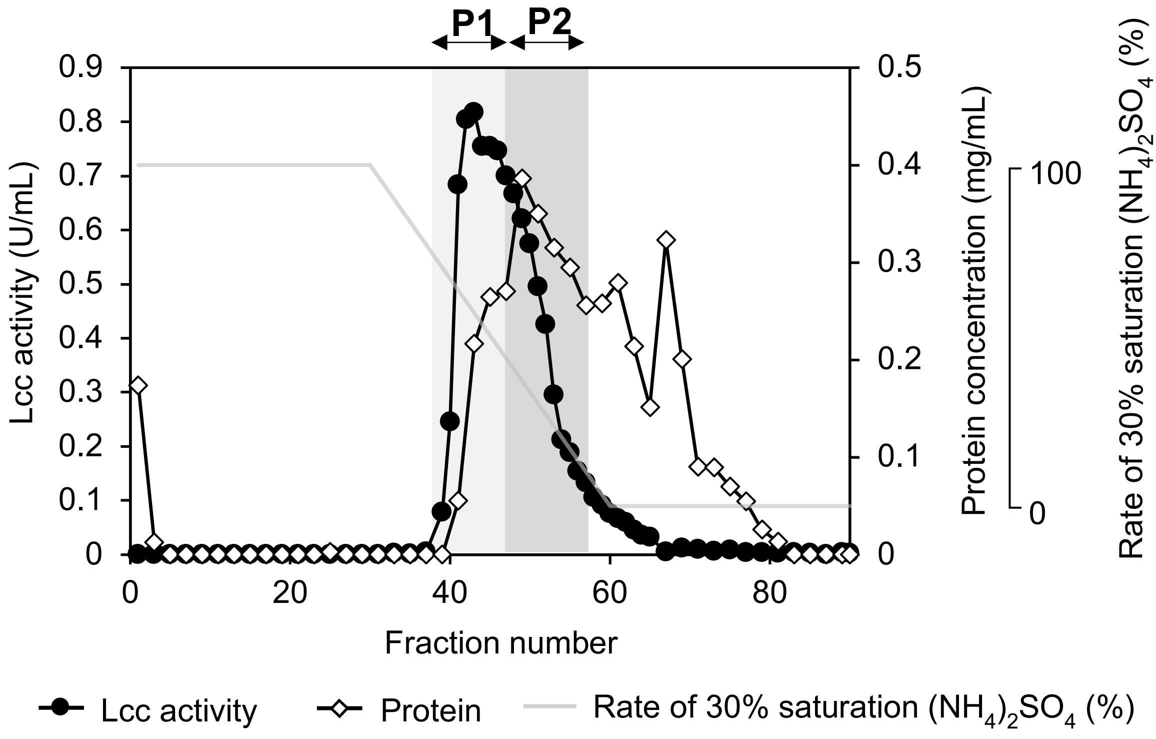

B

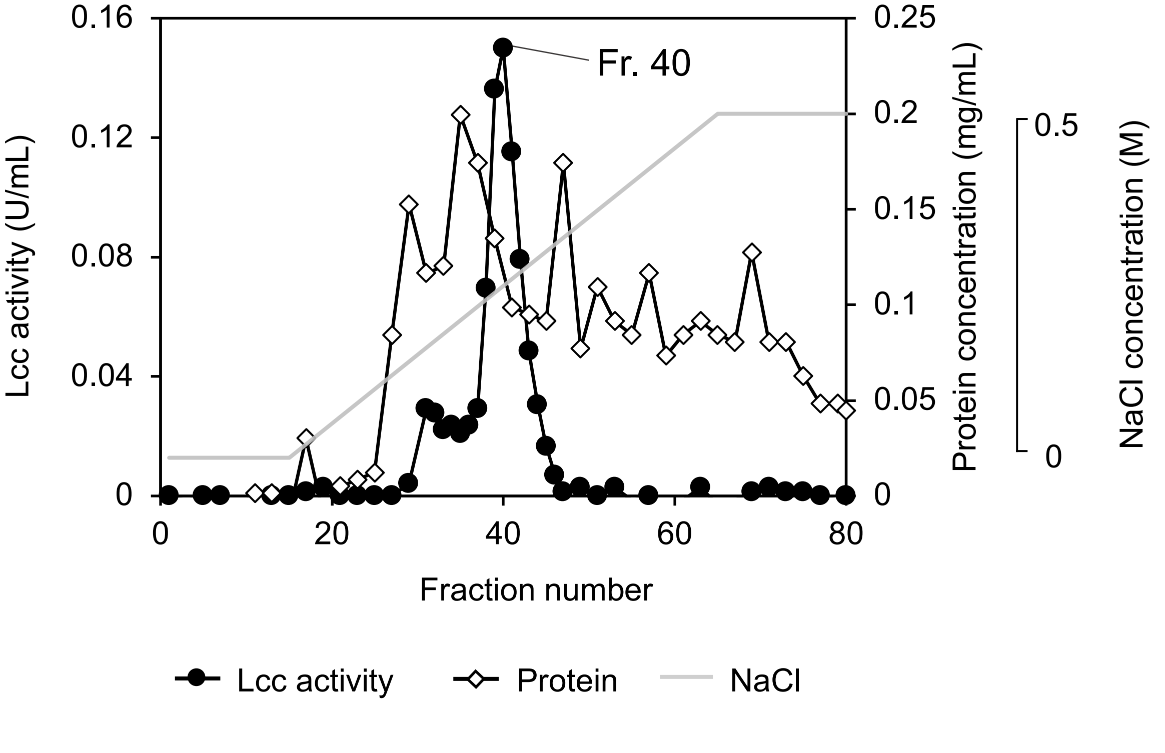

C

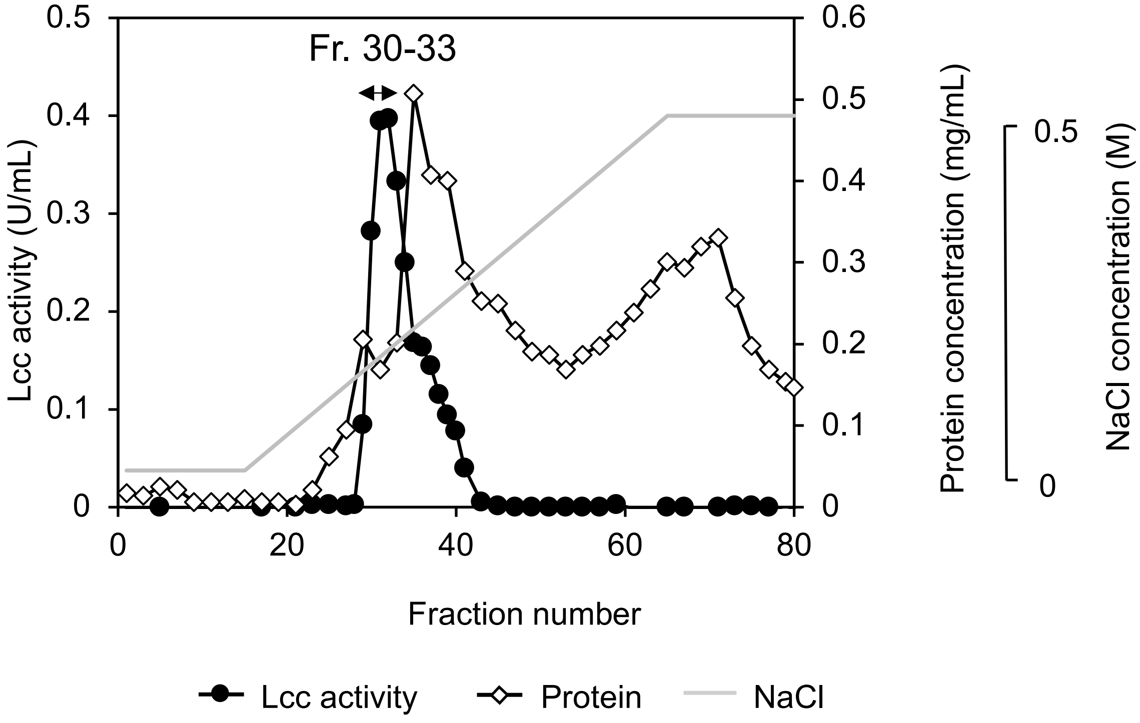

Figure S1. Laccase purification from the bottom part of the medium.

The Lcc purification was performed based on the enzymatic activity for ABTS. (A) Hydrophobic chromatogram of the crude enzyme on HiPrep™ Phenyl FF (high sub) 16/10 column. Arrows show recovered fractions, P1(Fr. 39-47) and P2 (48-57). (B) Anion exchange chromatogram of P1 on TOYOPEARL SuperQ-650M column. An active fraction, Fr. 40, was recovered. (C) Anion exchange chromatogram of P2 on TOYOPEARL SuperQ-650M column. Active fractions, Fr. 30-33, were recovered. The obtained active fractions were further applied to size exclusion chromatography, and the 3 partially purified fractions, P1-1 (from P1), P2-1 and P2-2 (from P2), were obtained.

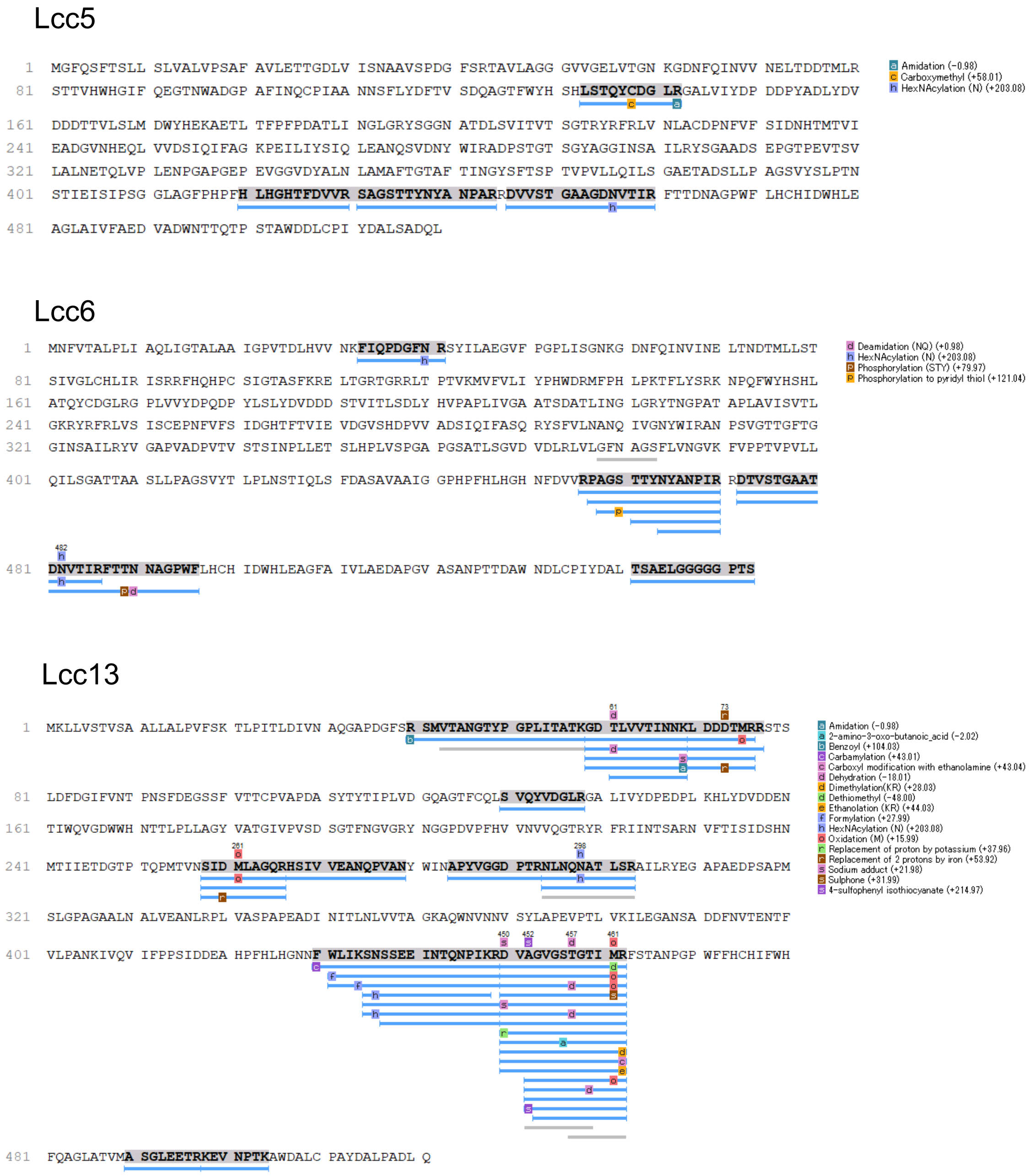

Figure S2. Coverage maps of peptide fragments of Lcc5, Lcc6 and Lcc13.

The SDS-PAGE bands were subjected to trypsin digestion and LC-MS/MS analysis and compared with the protein database of *Lentinula edodes* (Accession, PRJDB4944) (Sakamoto et al. 2017). *De novo* peptide sequencing was performed using PEAKS Studio v10. Homology searches were performed with BLAST (NCBI). Blue lines indicate digestible fragments analyzed with total sequence coverages of Lcc5 (10%), Lcc6 (11%) and Lcc13 (26%).
